# Incidence of influenza A(H3N2) virus infections in Hong Kong in a longitudinal sero-epidemiological study, 2009-2015

**DOI:** 10.1371/journal.pone.0197504

**Published:** 2018-05-24

**Authors:** Vivian W. I. Wei, Jessica Y. T. Wong, Ranawaka A. P. M. Perera, Kin On Kwok, Vicky J. Fang, Ian G. Barr, J. S. Malik Peiris, Steven Riley, Benjamin J. Cowling

**Affiliations:** 1 WHO Collaborating Centre for Infectious Disease Epidemiology and Control, School of Public Health, Li Ka Shing Faculty of Medicine, The University of Hong Kong, Hong Kong, Hong Kong Special Administrative Region, China; 2 Jockey Club School of Public Health and Primary Care, Faculty of Medicine, The Chinese University of Hong Kong, Shatin, Hong Kong Special Administrative Region, China; 3 Centre of Influenza Research, Li Ka Shing Faculty of Medicine, The University of Hong Kong, Hong Kong, Hong Kong Special Administrative Region, China; 4 Stanley Ho Centre for Emerging Infectious Diseases, The Chinese University of Hong Kong, Shatin, Hong Kong, Hong Kong Special Administrative Region, China; 5 Shenzhen Research Institute of the Chinese University of Hong Kong, Shenzhen, China; 6 WHO Collaborating Centre for Reference and Research, Melbourne, Victoria, Australia; 7 Department of Microbiology and Immunology, University of Melbourne, Melbourne, Victoria, Australia; 8 MRC Centre for Outbreak Analysis and Modelling, Department for Infectious Disease Epidemiology, Imperial College London, London, United Kingdom; Hokkaido University Graduate School of Medicine, JAPAN

## Abstract

**Background:**

Many serologic studies were done during and after the 2009 influenza pandemic, to estimate the cumulative incidence of influenza A(H1N1)pdm09 virus infections, but there are few comparative estimates of the incidence of influenza A(H3N2) virus infections during epidemics.

**Methods:**

We conducted a longitudinal serologic study in Hong Kong. We collected sera annually and tested samples from 2009–13 by HAI against the A/Perth/16/2009(H3N2) virus, and samples from 2013–15 against the A/Victoria/361/2011(H3N2) virus using the hemagglutination inhibition (HAI) assay. We estimated the cumulative incidence of infections based on 4-fold or greater rises in HAI titers in consecutive sera.

**Results:**

There were four major H3N2 epidemics: (1) Aug-Oct 2010; (2) Mar-Jun 2012; (3) Jul-Oct 2013; and (4) Jun-Jul 2014. Between 516 and 619 relevant pairs of sera were available for each epidemic. We estimated that 9%, 19%, 7% and 7% of the population were infected in each epidemic, respectively, with higher incidence in children in epidemics 1 and 4.

**Conclusions:**

We found that re-infections in each of the four H3N2 epidemics that occurred from 2010 through 2014 were rare. The largest H3N2 epidemic occurred with the lowest level of pre-epidemic immunity.

## Introduction

Influenza virus infections account for a significant annual disease burden [1, 2]. On average, influenza A(H3N2) epidemics tend to have the greatest impact on morbidity and mortality [3–5], particularly in elderly individuals [6, 7]. Influenza A(H3N2) viruses have also evolved more rapidly in more recent years, and between January 2000 and January 2015 there were 9 changes in the recommended A(H3N2) component of influenza vaccines compared to 4 changes in the A(H1N1) component including the A(H1N1)pdm09 pandemic virus [8]. Many serologic studies were done during and after the 2009 influenza pandemic, to estimate the cumulative incidence of influenza A(H1N1)pdm09 virus infections in the first [9, 10] and subsequent waves [5, 11], and the development of population immunity [12]. In comparison, there are relatively few population-based estimates of influenza A(H3N2) virus infections [5, 13]. Nor are there studies describing the role of population immunity in shaping consecutive epidemics of influenza A(H3N2).

In 2009 we began a population-based sero-epidemiologic study of influenza A(H1N1)pdm09 [14]. In that study we enrolled people from the general community using a systematic approach, and collected sera before and after the first wave of influenza A(H1N1)pdm09 virus infections. We subsequently extended this study through to 2015, collecting sera approximately once per year, and replacing participants that dropped out to maintain a sample size of between 800 and 1200 persons in each round of blood draws. In each study round, participants from previous rounds were invited to continue participating in the study. Participants that dropped out were replaced by new participants recruited via the same recruitment approach, i.e. random digit dialing. We used the hemagglutination inhibition (HAI) assay to measure antibody titers against circulating viruses. We have reported data from the first 4 rounds of blood draws, that covered two epidemics of A(H1N1)pdm09 and one epidemic of A(H3N2) [5]. Here we report data on influenza A(H3N2) virus infections in 4 consecutive epidemics in 2010, 2012, 2013 and 2014. The objective of this study is to describe the overall and age-specific incidence of H3N2 infections in Hong Kong, and to examine patterns in individual and population immunity as represented by HAI titers.

## Methods

### Study design

We designed a community-based open cohort study, with regular rounds of blood draws from cohort members, and regular replacement of participants that dropped out to maintain the sample size. The recruitment was on a household basis. At the start of the study in 2009, we used random digit dialing to approach people to participate in this study. People would be eligible to participate if they were at least 2 years of age and normally slept in the approached household for at least 5 nights per week. People who agreed to participate were invited to the study clinic where we collected baseline information on socio-demographics, any underlying medical conditions, and vaccination history, along with a 5ml clotted blood sample. The first round of blood draws was collected between July and September 2009 [14]. From November 2009 to February 2010 we invited participants to return to the study clinic for the second round of blood draws. We continued the study, with blood collected approximately one year apart, for 5 additional years around the start of each calendar year from 2011 to 2015. Therefore, our study comprised a total of seven rounds of blood draws (Rounds 1–7). In each round, in addition to blood samples, we administered the baseline questionnaire to new participants, and a brief questionnaire to continuing participants. Participants were compensated with HK$100 (around US$13) in the form of cash voucher and, since Round 3, HK$20 (around US$2.5) cash.

### Ethical approval

The study protocol was approved by the Institutional Review Board of the University of Hong Kong. We obtained written consent from participants 18 years of age or older. For participants aged 2 to 7 years, proxy written consent from parents or legal guardians was obtained. For participants aged 8 to 17 years, proxy written consent from parents or legal guardians was obtained in addition to their own written assent.

### Laboratory methods

We used the HAI assay to quantify the antibody titers against prevalent influenza A(H3N2) strains in the serum samples. Antibody titers were determined by testing serial 2-fold dilutions from 1:10 to 1:10240 in duplicate. The reciprocal of the highest dilution of serum that prevents complete hemagglutination in both duplicate wells was regarded as the antibody titer. We tested sera from Rounds 2–5 against the A/Perth/16/2009 (H3N2) virus, and those from Rounds 5–7 against the A/Victoria/361/2011 (H3N2) virus. We tested paired specimens from the same participants from consecutive rounds in parallel to ensure valid inferences of infections via rises in consecutive HAI titers.

### Statistical analysis

The primary outcome was serologic evidence of influenza A(H3N2) virus infection, which is defined as a four-fold or greater rise in HAI titers between consecutive paired sera. We identified major H3N2 epidemics from 2010 to 2015 using the weekly proportion of outpatient consultations with influenza-like illness multiplied by the weekly type/subtype-specific laboratory detection rates in the Public Health Laboratory Services Branch of the Centre for Health Protection [6, 15], defining the major H3N2 epidemics as those in which the proportion of samples positive for A(H3N2) exceeded 5% of all specimens submitted for at least 6 consecutive weeks. We estimated the cumulative incidence for each epidemic in four age groups (2-19y, 20-44y, 45-64y and ≥65y). Confidence intervals (CI) for the age-specific estimates were estimated by the exact binomial method; whereas those for the overall age group were estimated via the normal approximation and then age-standardized to the Hong Kong population in 2010. We excluded all participants with self-reported and unreported vaccination status in all analyses.

We estimated the rate of waning in HAI titers (in the absence of vaccination) against each virus strain by fitting log-linear regression models to HAI titers since infection in infected persons, in whom we had previously identified a ≥4 fold rise in HAI titers between paired sera and the titer value of the latter specimens being ≥20. We explored the waning rate since the time of infection, assuming that infections occurred in the peak of each community epidemic to determine the time since infection of the subsequent blood draws. We used logistic regression models to estimate the correlation of pre-epidemic HAI titers with protection against infection, where the reduction in the odds of infection risk at each HAI titer was estimated relative to an HAI titer <10. Having established the protection against serologic evidence of influenza virus infection, we used these risks to estimate the susceptibility index for each age group of participant (1 minus mean of reduction in the infection odds) at each HAI titer. The index for each epidemic was calculated as the average of the index values of all participants sampled before the corresponding epidemic, standardized to the population age structure, and ranged from 0 (i.e. population fully protected) to 1 (i.e. population fully susceptible to influenza virus infection). All statistical analyses were conducted using R version 3.3.2 (R Foundation for Statistical Computing, Vienna, Austria).

## Results

**Fig 1:**
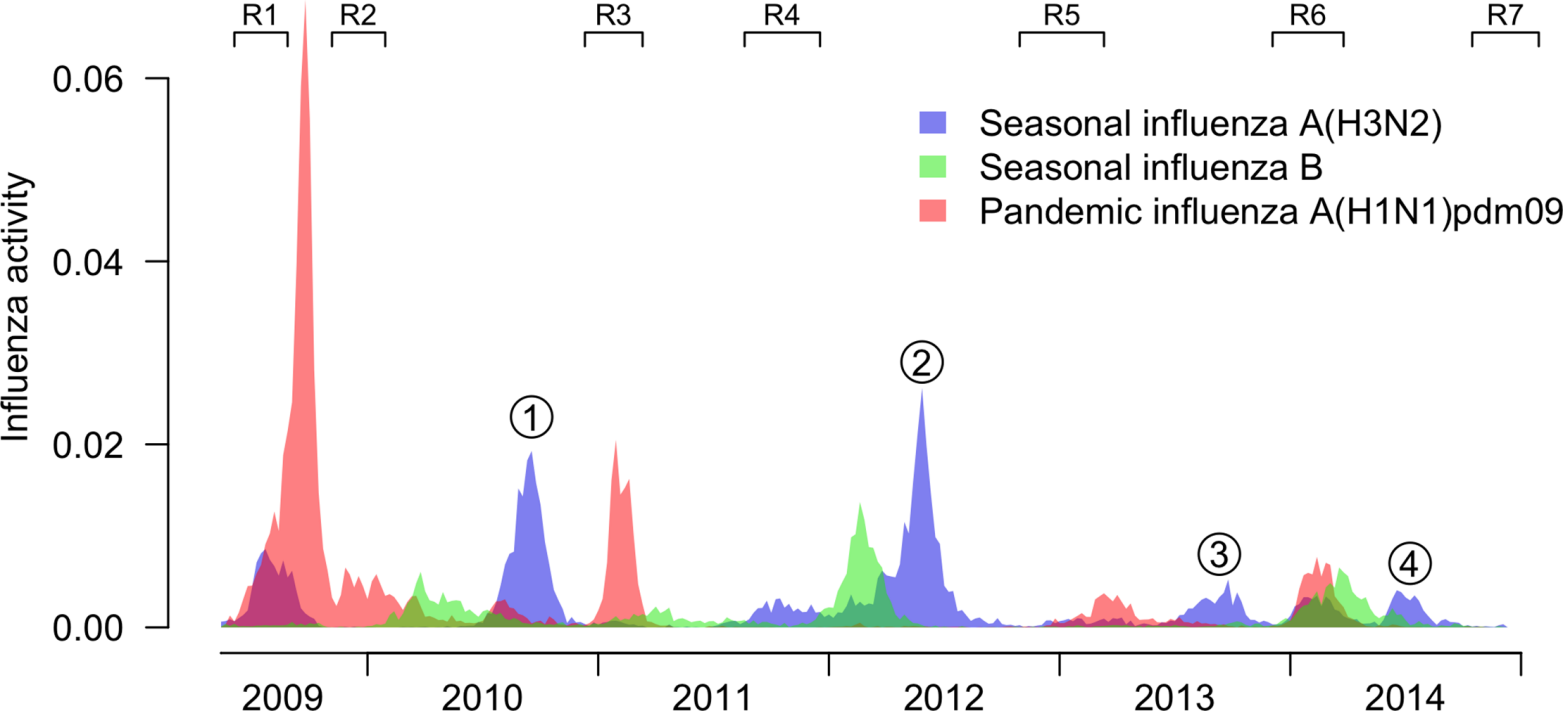
Timeline of our study rounds and community influenza virus activity. In total we collected blood in seven rounds, with each of the four numbered H3N2 epidemics being neatly bracketed by two consecutive rounds of blood draws. The y-axis shows weekly influenza virus activity in Hong Kong from 2009 to 2014, measured for each influenza type/subtype as the weekly proportion of outpatient consultations associated with influenza-like-illness in sentinel outpatient clinics multiplied by the weekly proportions of laboratory specimens testing positive for influenza A(H3N2), A(H1N1)pdm09 and B viruses respectively. For each type/subtype the activity level should correlate with incidence of infections within an epidemic, but changes in consultation behaviors between epidemics (e.g. in 2009/10) may also influence observed ‘activity’ levels.

The timeline of our study is shown in Fig 1, including the periods of the 7 rounds of sera collection, along with local surveillance data on influenza epidemics. During the study period, there were four major H3N2 epidemics and each was neatly bracketed by rounds of sera collection: (1) August-October 2010 (bracketed by Rounds 2 and 3); (2) March-June 2012 (bracketed by Rounds 4 and 5); (3) July-October 2013 (bracketed by Rounds 5 and 6); and (4) June-July 2014 (bracketed by Rounds 6 and 7). The four epidemics are indicated in Fig 1. The first two epidemics were dominated by A/Perth/16/2009-like viruses [16], while A/Victoria/361/2011-like viruses were predominant in the third and fourth epidemics.

For each of the four epidemics, we selected participants who provided sera in the rounds bracketing the corresponding epidemic, and inferred the cumulative incidence of infections in that epidemic based on analyses of the relevant pairs of sera. Between 516 and 619 relevant pairs of sera were available for each epidemic (Table 1). Participants who self-reported receipt of vaccination before or during the corresponding influenza season were excluded from the analysis.

**Table 1:** Characteristics of participants with paired sera available for each influenza A(H3N2) epidemic studied.

	Epidemic 1	Epidemic 2	Epidemic 3	Epidemic 4
	(n = 516)	(n = 558)	(n = 619)	(n = 585)
**Age (years)**				
2–19	516 (100%)	558 (100%)	619 (100%)	585 (100%)
20–44	57 (11%)	24 (4%)	32 (5%)	35 (6%)
45–64	133 (26%)	95 (17%)	124 (20%)	106 (18%)
≥65	259 (50%)	359 (64%)	330 (53%)	298 (51%)
	67 (13%)	80 (14%)	133 (21%)	146 (25%)
**Sex**				
Male	203 (39%)	228 (41%)	242 (39%)	229 (39%)
Female	313 (61%)	330 (59%)	377 (61%)	356 (61%)
**Self-reported seasonal** **influenza vaccination**				
Yes	122 (24%)	83 (15%)	139 (22%)	149 (25%)
No	392 (76%)	466 (84%)	478 (77%)	435 (74%)
Unknown	2 (0%)	9 (2%)	2 (0%)	1 (0%)
**Dropout**	302 (59%)	163 (29%)	147 (24%)	NA

NA = not available

Estimates of the cumulative incidence of A(H3N2) infections in each epidemic are shown in Table 2. Overall, 7%-19% of the population were infected in each epidemic. However, epidemic 2 (Mar-Jun 2012) was substantially larger than the other epidemics with an estimated overall attack rate of 19% (95% CI: 14%, 24%). The cumulative incidence of infection was generally the highest among children, but similar across different age groups in epidemics 2 and 3. Re-infections were rare, with evidence in a small number of participants followed in particular pairs of epidemics. Of the 707 unvaccinated participants who provided samples covering epidemics 1 and 2, we identified 0 (0%) with evidence of infection in both of those epidemics i.e. re-infection. Similarly, we identified 1/706 (0.1%) for epidemics 1 and 3, 0/689 (0%) for epidemics 1 and 4, 2/639 (0.3%) for epidemics 2 and 3, 2/677 (0.3%) for epidemics 2 and 4, and 0/576 (0%) for epidemics 3 and 4.

**Table 2:** Cumulative incidence of infection and corresponding 95% confidence intervals in each of the four influenza A(H3N2) epidemics during the study period.

Age (years)	Epidemic 1 (Aug-Oct 2010)	Epidemic 2 (Mar-Jun 2012)	Epidemic 3 (Jul-Oct 2013)	Epidemic 4 (Jun-Jul 2014)
2–19	0.11 (0.04, 0.25)	0.21 (0.07, 0.42)	0.04 (0.00, 0.18)	0.17 (0.06, 0.36)
20–44	0.05 (0.02, 0.11)	0.15 (0.08, 0.24)	0.06 (0.03, 0.13)	0.04 (0.01, 0.10)
45–64	0.10 (0.06, 0.15)	0.23 (0.18, 0.28)	0.07 (0.04, 0.10)	0.04 (0.02, 0.07)
≥65	0.17 (0.05, 0.39)	0.20 (0.09, 0.34)	0.14 (0.06, 0.26)	0.07 (0.01, 0.18)
Overall*	**0.09 (0.06, 0.13)**	**0.19 (0.14, 0.24)**	**0.07 (0.04, 0.10)**	**0.07 (0.04, 0.10)**

*Standardized by age to the Hong Kong population in 2010.

**Fig 2:**
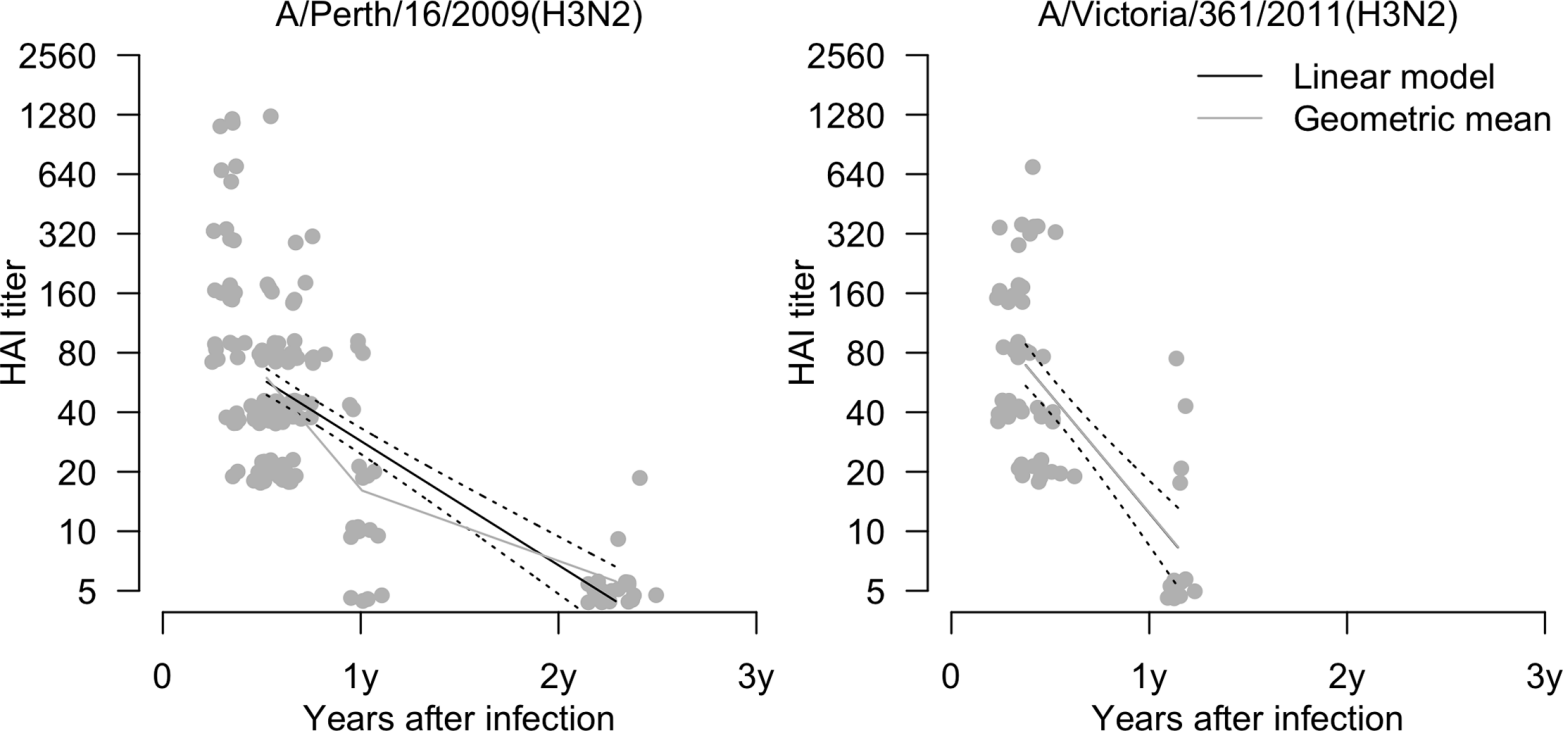
Declines in HAI titers against influenza A/Perth/16/2009(H3N2) and A/Victoria/361/2011(H3N2) virus after infection. In each panel the black regression lines indicate the rates of antibody waning from a fitted log-linear model, and the grey lines indicate the geometric mean titers at the center of each time point that sera were collected. There were 126 and 44 participants infected against A/Perth/16/2009(H3N2) and A/Victoria/361/2011(H3N2) respectively.

**Fig 3:**
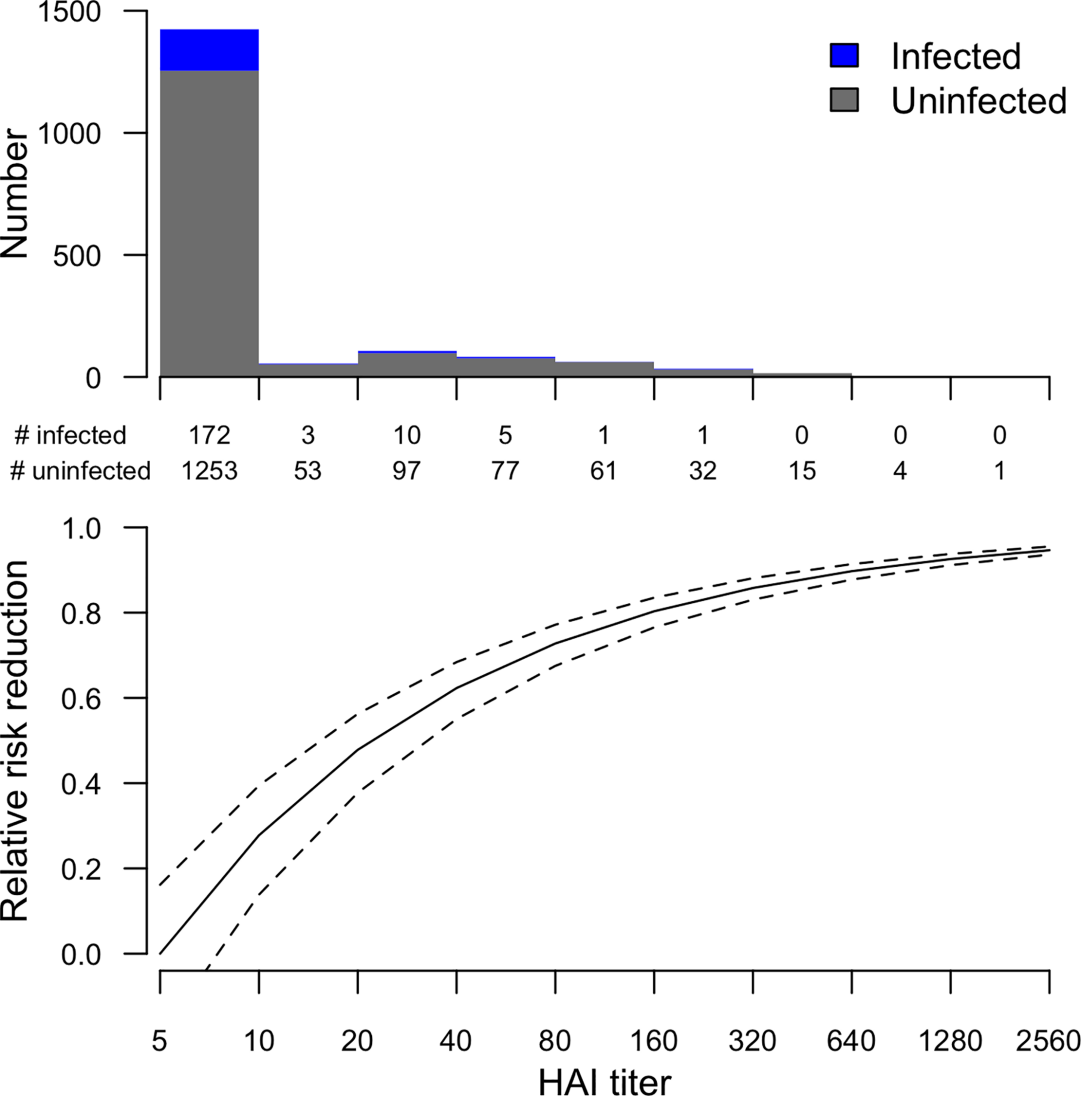
Correlation between HAI titers and protection against influenza A(H3N2) virus infection. The upper panel shows the number of uninfected and infected persons in each pre-epidemic titer range. The lower panel shows the estimated degree of protection associated with higher pre-epidemic titers, calculated as the relative risk reduction compared with the risk at a pre-epidemic HAI titer <10.

We estimated that 2-fold declines in HAI titers occurred after means of 175 (95% CI: 149, 211) days for A/Perth/16/2009(H3N2), and after means of 92 (95% CI: 74, 121) days for A/Victoria/361/2011(H3N2) (Fig 2). Higher HAI titers corresponded to a greater reduction in the risk of A(H3N2) infection, and an HAI titer of 40 correlated with 62% (95% CI: 55%, 68%) protection against infection (Fig 3). We were not able to stratify this analysis by strain and by epidemic due to the low number of infected cases with pre-epidemic titers >10.

**Fig 4:**
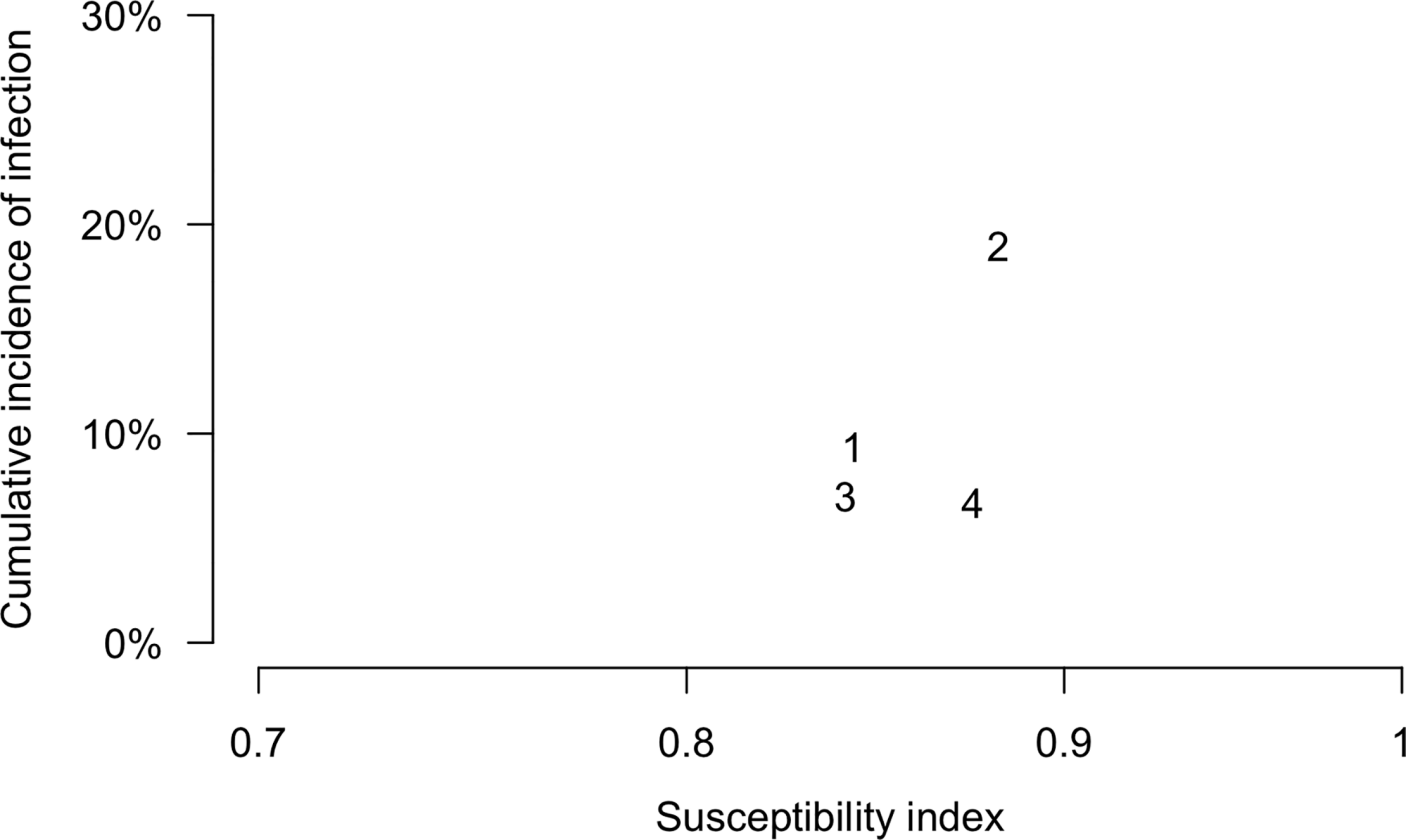
Comparison between susceptibility and age-standardized cumulative incidence of infection in four influenza A(H3N2) epidemics. The susceptibility index was calculated as 1 minus relative risk reduction compared with the risk at per-epidemic HAI titer <10. The cumulative incidence of infection was standardized by age to the Hong Kong population in 2010. Each text 1 to 4 corresponds to epidemics 1 to 4 respectively.

We estimated a susceptibility index for each epidemic based on the distribution of pre-epidemic titers and the protection curve shown in Fig 3. Compared with epidemics 1, 3 and 4, individuals were more susceptible to infection in epidemic 2 (susceptibility index = 0.88), and that epidemic had the highest overall cumulative incidence of infection of 19% (Fig 4).

## Discussion

We conducted a longitudinal serologic study over five years, allowing us to estimate the cumulative incidence of influenza A(H3N2) virus infections in four consecutive epidemics in Hong Kong. We found that 7% - 19% of the population were infected in each of these epidemics, based on the standard definition of a four-fold rise or greater in homologous titers, and the incidence of infection was generally higher in children (Table 2). There was a higher cumulative incidence of infections in children (2–19 years) and elderly individuals (65 years or greater) compared with other adults (20–64 years) (Table 2). Hong Kong is a densely populated city, where the observation here about difference in infection in age groups could be due to social mixing patterns [17, 18] and transmission dynamics [5, 14].

While we excluded vaccinated persons when estimating the cumulative incidence of infection, influenza vaccination coverage has been very low in Hong Kong, around 5%-10% overall, with coverage of around 40% in elderly individuals [19], 30% in healthcare workers [20], and low in most other groups including children. If we had accounted for coverage of 40% in elderly individuals with effectiveness of around 40% against H3N2 infection in these years [16], our estimates of the cumulative incidence of natural infection in that age group would have been 16% lower. Vaccination coverage of 5%-10% in the population as a whole, with effectiveness of 50% on average, would have had very little impact on our estimates of the overall cumulative incidence of infections.

Our estimates of the rate of antibody waning in HAI titers since infection in infected persons were similar to those in another study [21], where the mean time to 2-fold decline in HAI titer was 92–175 days (Fig 2). In addition, antibody declined more quickly in participants infected with A/Victoria/361/2011-like (in epidemics 3 and 4) viruses compared with participants infected with A/Perth/16/2009-like viruses (in epidemics 1 and 2). We identified a correlation of pre-epidemic HAI titers with protection against infection. The classic study by Hobson [22], and other studies [23–25], have identified a correlation of titers of 40 with 50% protection against infection, which is somewhat less than the 62% (95% CI: 55%, 68%) protection that we estimated was conferred by a pre-epidemic titer of 40 in our study. However, in contrast to most recent studies, our outcome was serologic evidence of influenza virus infection, not laboratory-confirmed influenza virus detection.

We noted that the level of population immunity varied before each of the four epidemics, and we created a single statistic to represent population immunity by combining information on titer distributions and the protection associated with those titers. Using this summary statistic of immunity, we found that the largest A(H3N2) epidemic occurred with the lowest level of pre-epidemic immunity (Fig 4). According to local surveillance data, this epidemic was dominated by A/Perth/16/2009-like viruses [16], which had circulated since 2009. However, despite circulation for 3 years, population immunity had declined and was lower before epidemic 2 (2012) than it was before epidemic 1 (2010) in our study (Fig 4). In further work, it would be of interest to examine alternative measures of population immunity and determine which could provide a best representation of the vulnerability of a population to a larger epidemic. For example, the level of immunity in children might be a particularly important aspect of the risk to the population as a whole for severe epidemics.

There are a number of limitations of our study. First, we used serologic data to infer the occurrence of A(H3N2) infections based on 4-fold or greater rises in HAI titers, but this approach may have missed some infections [26, 27]. Some A(H3N2) infections are associated with low rises in HAI titers [26], and therefore we may have underestimated the cumulative incidence of infections. Second, we relied on self-reported vaccination status, and we investigated the serologic data for evidence of rises in HAI titers against A(H1N1) and A(H3N2) all together which might be indicative of vaccination rather than infection. There were no participants in epidemics 1 to 4 having such a result. These were excluded from the estimation of cumulative incidence of infections, but we may have failed to identify vaccinations in a small number of other participants. Third, our participants may not be representative of the general population because of biases in participation. We did enroll using random digit dialing to select potential participants at random from the population, but the response rate was low. Self-reported vaccination coverage in our participants was considerably higher than the general population, and we excluded vaccinated participants when estimating the cumulative incidence of infection. Fourth, the study rounds of sera did not neatly bracket each A(H3N2) epidemics and we were not able examine the two small A(H3N2) epidemics in 2011 and early 2014, or A(H3N2) infections that occurred outside the four major epidemics that we identified in Fig 1. Epidemics overlapping study rounds may underestimate or overestimate the number of infections in the epidemic following or preceding the study rounds, respectively. We are actively developing statistical techniques that can characterize these smaller epidemics with these irregular follow-up timings. Finally, we did not examine A(H1N1) or B in this study, because our focus was on A(H3N2), but cross reactive antibodies to these strains may have affected titers to A(H3N2).

In conclusion, we estimated that 7% - 19% of the Hong Kong population were infected in each of the four H3N2 epidemics from 2010 to 2014, and re-infections in these epidemics were rare. With the single measure of pre-epidemic immunity which we created, we found that the largest H3N2 epidemic occurred with the lowest level of pre-epidemic immunity.
